# The diagnostic value of C-X-C motif chemokine 8 in elderly patients with bacterial upper respiratory tract infections and its correlation with prognosis

**DOI:** 10.3389/fmed.2025.1694303

**Published:** 2026-02-02

**Authors:** Lei Yu, Youtao Hu, Jianbin Sun, Xinguang Liu

**Affiliations:** 1 Department of Clinical Laboratory, The Third Hospital of Wuhan, Wuhan, Hubei, China; 2 Guangdong Medical University, Zhanjiang, Guangdong, China

**Keywords:** respiratory tract infections, interleukin-8, elderly, severity, prognosis

## Abstract

**Objective:**

This study aimed to further investigate the correlation between C-X-C motif chemokine 8‌ (CXCL8) and the prognosis of bacterial upper respiratory tract infections (BURTIs) in the elderly population (≥65 years) and to evaluate its potential as a clinical biomarker in this specific high-risk cohort.

**Methods:**

A total of 58 elderly patients with BURTIs admitted to our hospital between January 2023 and June 2023 (observation group) and 42 healthy individuals (control group) who underwent physical examinations concurrently were selected as the research subjects. Peripheral blood CXCL8 levels were measured in both the observation and control groups at admission and reassessed in the observation group after treatment to determine differences in CXCL8 between groups and changes in CXCL8 before and after treatment in the observation group. The correlation of CXCL8 with BURTI clinical effectiveness was analyzed. Subsequently, BURTI patients were followed up for 1 year, and the effect of post-treatment CXCL8 expression on the prognostic recurrence of BURTIs was evaluated.

**Results:**

The CXCL8 in the observation group was higher than that in the control group (89.67 ± 8.33 pg./mL *vs.* 71.20 ± 10.88 pg./mL) and decreased after treatment (*p* < 0.05). Patients with recurrence showed higher CXCL8 levels than those without (78.44 ± 8.84 pg./mL *vs.* 69.67 ± 5.51 pg./mL, *p* < 0.05). According to the receiver operating characteristic (ROC) curve analysis, CXCL8 exhibited excellent effects on the occurrence and prognostic recurrence of BURTIs (AUC = 0.788).

**Conclusion:**

CXCL8 is elevated in elderly patients with BURTIs, demonstrating good evaluation effects on the occurrence and prognostic recurrence of BURTIs. However, these findings position CXCL8 as a promising biomarker for guiding personalized treatment in elderly BURTI patients, though further validation and integration with clinical scores are needed to improve risk stratification.

## 1 Introduction

Bacterial upper respiratory tract infections (BURTIs) are one of the most common infectious diseases in the world and can occur in all age groups (1). According to the statistics of the World Health Organization, the number of new BURTI cases worldwide reached approximately 480 million in 2019, with a standard incidence rate of 6,295 per 100,000 (2). Among them, cases are most common in children under 10 years of age and in older adults over 65 years of age, which is closely related to poor immune function in such patients (3). As a result, there has always been a lack of dynamic evaluation indicators for BURTIs in clinical practice, making it difficult to timely understand the progression of the patient’s condition.

Recently, studies have attempted to assess the occurrence of BURTIs by procalcitonin (PCT) (4) and interleukins (IL)-6 (5). However, both PCT and IL-6 have been confirmed to be related to a variety of inflammatory and stress responses in the human body (6, 7); thus, the sensitivity of evaluating BURTIs is not significant. C-X-C motif chemokine 8‌ (CXCL8), a member of the IL family, is selected because its chemokine receptors have a cellular chemotaxis effect on neutrophils to regulate inflammatory responses (8). CXCL8 holds distinct advantages: its chemotactic activity directly reflects neutrophil recruitment dynamics—a hallmark of BURTI pathogenesis (9). Moreover, CXCL8’s role in modulating T-cell glycolipid metabolism suggests potential links to immune evasion mechanisms in chronic infections (10), in which CXCL8 may also have important application potential. While CXCL8 has been studied in viral infections such as COVID-19—where blood levels increase but bronchial levels decrease due to CXCR2 dysregulation (11)—direct evidence in BURTIs in the elderly remains limited. BURTIs in the elderly—such as streptococcal pharyngitis or bacterial sinusitis—pose significant diagnostic challenges due to atypical presentations (12). While viral studies inform CXCL8 biology, our focus is on BURTIs, in which neutrophilic inflammation is paramount.

Although CXCL8 has been extensively studied in BURTIs, its role in elderly patients (>65 years)—a population with weakened immune responses and atypical clinical presentations—remains poorly understood. At the same time, there is still a lack of direct clinical evidence for the diagnosis and prognosis evaluation of CXCL8 in BURTIs. This study aims to systematically investigate CXCL8 dynamics in elderly individuals with BURTIs. At the same time, this study verifies the effect of CXCL8 in the diagnosis and prognosis evaluation of elderly BURTIs and provides a direct reference for clinical practice. These results have important reference significance for the diagnosis and treatment of elderly BURTIs in the future.

## 2 Information and methodology

### 2.1 Study population

We conducted a prospective analysis. From January 2023 to June 2023, 58 elderly BURTI patients (observation group) and 42 age-matched (≥65 years old) healthy individuals (control group) with no recent history of acute respiratory infection (e.g., within the past 3 months) and normal physical examination results (including blood routine, C-reactive protein, and other inflammatory indicators) were enrolled. The number of cases was determined based on calculations using G-Power 3.1 software (two-tailed test, effect = 0.5, *α* = 0.05, power = 0.95, minimum sample size of 42) and screening for the inclusion and exclusion criteria (some of the minimum sample size was added to account for the possibility of dropout). This study has been approved by the Ethics Committee of our hospital and is carried out in strict accordance with *the Declaration of Helsinki*. All the study subjects and the research team members who collected the data were unaware of the group assignments of the study subjects.

### 2.2 Eligibility and exclusion criteria

Observation group: The patients included (≥65 years old) had complete case data and were confirmed by a bacteriological diagnosis of respiratory secretions (BURTIs), with voluntary participation in this research and informed consent provided. Those excluded were in line with any of the following conditions: acute suppurative tonsillitis, acute sinusitis, lower respiratory tract infections, whooping cough, tuberculosis, or measles; pregnant and lactating patients; allergic constitution, or multiple drug allergies; malignant tumors, cardio-cerebrovascular diseases, liver and kidney dysfunction, or other serious primary diseases; and mental diseases. Control group: Patients (≥65 years old) who had complete case data, no history of acute respiratory infection within the past 3 months, no current symptoms suggestive of infection (e.g., fever, cough, or sputum), and normal results on physical examination and routine laboratory tests (including a complete blood count and C-reactive protein) were included; they were fully informed and voluntarily participated in this research, with informed consent obtained. The exclusion criteria were the same as those in the observation group. To minimize recall bias regarding recent respiratory infections, we verified the absence of acute respiratory infection in the past 3 months through a medical record review and a standardized questionnaire administered by clinicians. The questionnaire included specific symptoms (such as fever, cough, and sputum) and healthcare visits related to respiratory illnesses, with cross-checking against electronic health records when available.

### 2.3 Treatment methods

After admission, BURTI patients received anti-infective therapy against pathogens, while maintaining water–electrolyte acid–base balance, correcting hypoalbuminemia, and providing nutritional support. Besides, atomization, postural drainage, and chest physiotherapy were provided as appropriate. Patients with hypoxemia received oxygen therapy to maintain blood oxygen saturation above 90%; those requiring respiratory support were provided timely mechanical ventilation to restore effective ventilation and improve oxygenation.

### 2.4 Effectiveness evaluation

Clinical criteria were used to assess effectiveness, including the resolution of symptoms (such as cough and fever). Microbiological: culture clearance (post-treatment swab). Biomarker: CRP < 10 mg/L and PCT < 0.25 ng/mL. Cure was defined as fulfillment of all three criteria; improvement was defined as the achievement of clinical effectiveness plus the fulfillment of one objective criterion; failure indicated no change in any of the three criteria.

### 2.5 Follow-up for prognosis

All BURTI patients had at least 1 year of prognostic follow-up or hospital re-examinations, with an interval of no more than 2 months between each review or follow-up visit, and the recurrence of BURTIs was recorded. BURTI recurrence is defined as the recurrence of clinical symptoms of BURTIs in patients and a positive sputum culture.

### 2.6 Sample collection and testing

Fasting venous blood, which was collected from both the observation and control groups at admission and from the observation group after treatment, was left at room temperature for 30 min and centrifuged to obtain the serum. Levels of CXCL8 were measured using a magnetic particle chemiluminescence immunoassay (fully automated chemiluminescence immunoanalyzer, Hotgen, C2000).

### 2.7 Endpoints

Differences in CXCL8 between groups and changes in CXCL8 in the observation group before and after treatment were observed to clarify the correlation of CXCL8 with BURTI severity, clinical effectiveness, and prognostic recurrence and to analyze the evaluation effect of CXCL8 on the condition of elderly BURTI patients.

### 2.8 Statistical analysis

This study used SPSS24.0 for statistical analysis and GraphPad Prism 9 for graphic drawing. Categorical variables such as patient’s sex and family disease history were expressed by [*n* (%)], and the chi-square tests were used for inter-group comparisons. Continuous variables such as patient’s age and CXCL8 expression were described as (^−^*χ* ± s), with inter-group comparisons made by independent sample t-tests. The diagnostic value was determined by receiver operating characteristic (ROC) curves, and the area under curve (AUC) was used to determine the diagnostic effect. The cutoff value, sensitivity, and specificity were determined according to the maximum value of the Youden index. The recurrence rate was calculated and compared using the Kaplan–Meier method and the log-rank test, respectively. A significance level of *p* of <0.05 was used in all analyses.

## 3 Results

### 3.1 The observation and control groups were similar in general data

First, general data such as age, sex, and family disease history were compared between the two groups, with no statistical significance identified (*p* > 0.05), confirming comparability (Table 1).

**Table 1:** Comparison of clinical data.

Groups		Control (*n* = 42)	Observation (*n* = 58)	*t* (or *c*^2^)	*p*
Age		72.86 ± 4.21	72.71 ± 4.93	0.212	0.832
BMI (kg/m^2^)		23.05 ± 1.99	23.39 ± 1.72	0.922	0.359
Duration of disease (d)		–	28.57 ± 7.60	–	–
Sex	Male/female	26 (61.90)/16 (38.10)	34 (58.62)/24 (41.38)	0.11	0.741
Smoking	Yes/no	15 (35.71)/27 (64.29)	24 (41.38)/34 (58.62)	0.329	0.567
Severity	Mild/moderate/severe	–	16 (27.59)/33 (56.90)/9 (15.52)	–	–
Combined diabetes mellitus	Yes/no	17 (40.48)/25 (59.25)	26 (44.83)/32 (55.17)	0.188	0.664
Combined hypertension	Yes/no	15 (35.71)/27 (64.29)	27 (46.55)/31 (53.45)	1.175	0.279

### 3.2 Diagnostic value of CXCL8 in BURTIs

**Figure 1:**
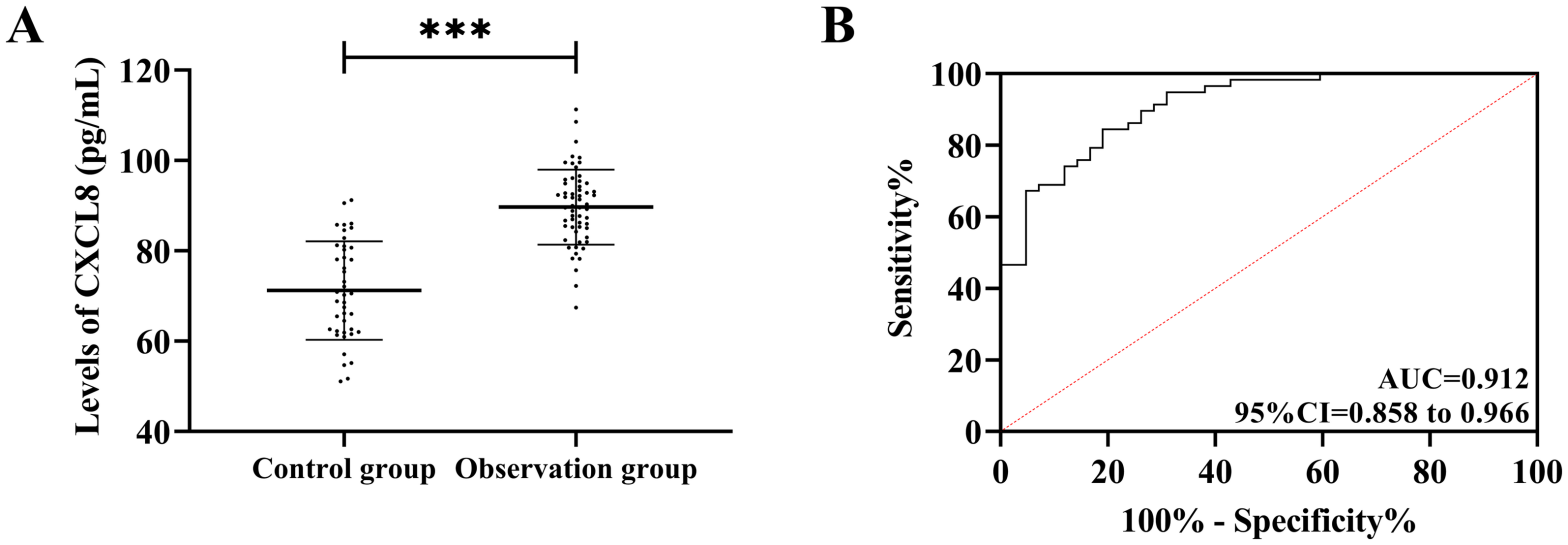
Comparison of CXCL8. **(A)** Comparison of CXCL8 in observation and control groups. **(B)** ROC curve for the CXCL8 diagnosis of BURTI occurrence. ***p < 0.001.

After testing, CXCL8 in the observation group was 89.67 ± 8.33 pg./mL, higher compared to the control group (71.20 ± 10.88 pg./mL) (t = 9.614, *p* < 0.05), suggesting that CXCL8 is upregulated in BURTIs. The ROC curve analysis revealed that, when CXCL8 was >81.57 pg./mL, its sensitivity and specificity in diagnosing BURTIs were 84.48 and 90.95%, respectively (AUC = 0.912, 95%CI = 0.858–0.966, *p* < 0.001) (Figure 1).

### 3.3 Correlation of CXCL8 with clinical effectiveness of BURTI patients

**Figure 2:**
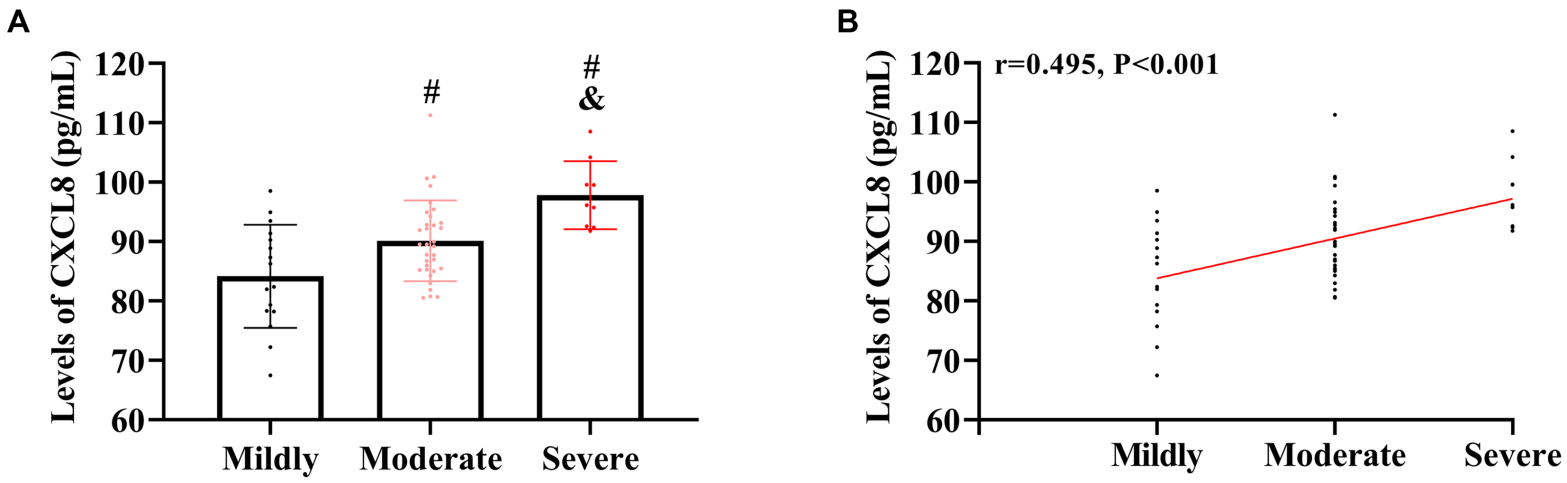
Relationship between CXCL8 and clinical outcomes, ^***^p < 0.001. **(A)** Changes in CXCL8 before and after treatment. **(B)** Comparison of CXCL8 in BURTI patients with different effectiveness (after treatment), * denotes *p* < 0.05 compared with cured patients, # denotes *p* < 0.05 compared with marked effectiveness patients, and & denotes *p* < 0.05 compared with effectiveness patients.

The level of CXCL8 in the observation group after treatment was 71.78 ± 7.35 pg./mL, which was lower than that before treatment (*t* = 12.342, *p* < 0.05). Among patients with different curative effects, CXCL8 levels were lowest in cured patients [(64.00 ± 2.26) pg./mL] after treatment, followed by markedly effective [(70.68 ± 4.94) pg./mL] and effective patients [(72.42 ± 5.08) pg./mL], while ineffective patients [(82.41 ± 6.80) pg./mL] showed the highest CXCL8 levels after treatment (*F* = 24.318, *p* < 0.05) (Figure 2).

### 3.4 Relationship between CXCL8 and prognosis of BURTIs

**Figure 3:**
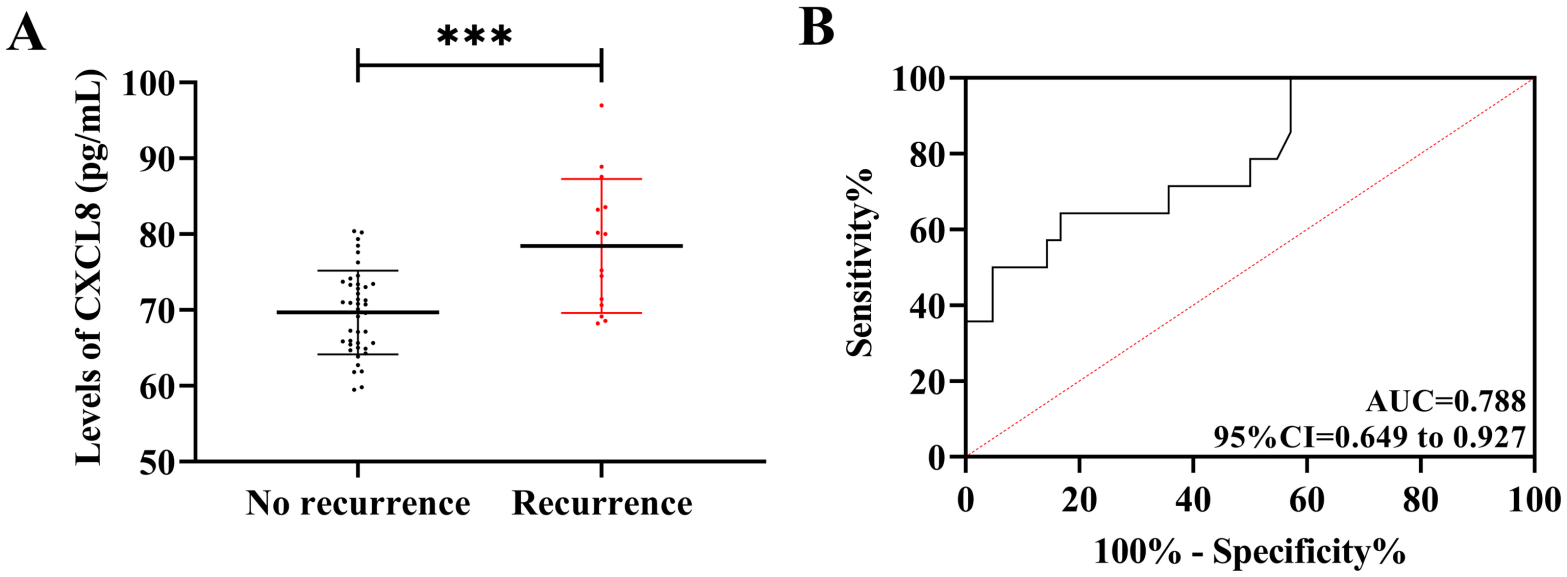
Relationship between CXCL8 and the prognostic recurrence of BURTIs. **(A)** Comparison of CXCL8 between prognostic relapsed and non-relapsed patients (after treatment). **(B)** ROC curves for CXCL8 diagnosis of prognostic relapse in BURTIs (after treatment). ***p < 0.001.

In the follow-up of prognosis, we successfully tracked 56 patients in the observation group (median follow-up was 7 months), of which 14 patients had BURTI recurrence, with a 1-year overall recurrence rate of 25.00%. By comparison, it was found that the CXCL8 levels in patients with recurrence after treatment were higher than those without recurrence [(78.44 ± 8.84) pg./mL *vs.* (69.67 ± 5.51) pg./mL] (*t* = 4.392, *p* < 0.05). The ROC curve analysis further showed that, when the CXCL8 was above 74.32 pg./mL after treatment, the sensitivity and specificity for predicting 1-year prognostic recurrence in BURTI patients were 64.29 and 83.33%, respectively (AUC = 0.788, 95%CI = 0.649–0.927, *p* = 0.001) (Figure 3).

## 4 Discussion

The incidence of BURTIs ranks first in acute infectious diseases, and the occurrence and progression are mostly related to region, environment, climate, and patient age, especially in infants, young children, older adults, and immunocompromised patients, which is also an important cause of their death (13). Owing to the multifactorial pathogenesis of BURTIs, clinical symptoms and routine examination are often insufficient for a definitive diagnosis. Blind medication may exacerbate the condition or cause further delays in effective treatment. However, conventional etiological diagnosis not only takes a long time but also has a long waiting time due to a variety of external factors, which adversely affect the accuracy of diagnostic results, further leading to delays and is therefore unfavorable for subsequent treatment and prognosis of patients (14). Building upon the established knowledge of CXCL8’s role in inflammation and infection, this study specifically investigated its profile in elderly patients with bacteriologically confirmed respiratory tract infections. Our results confirm and extend prior observations by demonstrating that CXCL8 levels are significantly elevated in this specific patient group compared to healthy elderly controls. Moreover, CXCL8 has a good application value in the disease assessment of BURTIs, which lays a foundation for developing new clinical diagnosis and treatment schemes for BURTIs in the future.

First, comparing CXCL8 levels between BURTI patients and healthy individuals, it was found that CXCL8 levels were significantly elevated in BURTI cases, suggesting the possible involvement of CXCL8 in the occurrence and development of BURTIs. This finding is consistent with the research results of Govoni et al. (15) in exploring CXCL8 expression in chronic obstructive pulmonary disease. CXCL8 is primarily produced by monocytes–macrophages, with a molecular weight of approximately 8 kD and a major active form of 72 amino acids. Other cells, such as fibroblasts, epithelial cells, endotheliocytes, and hepatocytes, can also produce CXCL8 under suitable stimulation conditions (16). The amino acid sequence of CXCL8 is highly homologous to many inflammatory cytokines and belongs to the same family. The mechanism is to attract and activate neutrophils, orient themselves to the site of response, and release a series of inflammatory products (17). Therefore, the impact of CXCL8 on inflammatory diseases such as Crohn’s disease and osteoarthritis has received widespread clinical attention (18, 19). We hypothesized that hypoxic response in airway tissue post-BURTIs stimulates eosinophils to secrete CXCL8, based on the known role of hypoxia in neutrophilic inflammation. However, hypoxic response may not be universal in all BURTI cases—it is more likely in severe infections with significant airway obstruction. Alternatively, mast cell-derived mediators (e.g., LTB4 and TNF-*α*) in hypoxic preconditioning could promote eosinophil recruitment and CXCL8 secretion (20). Thus, while hypoxia is a plausible pathway, CXCL8 elevation in BURTIs may also reflect complex interactions involving mast cells and other immune cells. Future studies should directly measure hypoxia indicators (e.g., PaO2) to validate their role. Subsequently, CXCL8 chemoattracts neutrophils to accumulate in the inflammatory site, which, with the participation of various inflammatory pathways, causes damage to surrounding tissues and sustained development of inflammation, promoting the development of BURTIs (21). In a study on articular chondrocytes by Jacob et al., the level of CXCL8 gradually increases with the aging of articular chondrocytes (22), consistent with our observations. In the prognostic follow-up, we found higher CXCL8 expression in post-treatment BURTI patients who experienced recurrence compared to those who did not, which is presumably due to the worse pathological condition of patients with recurrent BURTIs. While CXCL8 elevation initially promotes neutrophil recruitment, sustained high levels—as noted in severe BURTIs—can induce CXCR2 desensitization and internalization, leading to migratory arrest and neutrophil accumulation in microvasculature. This phenomenon, which is well-documented in sepsis and COVID-19 (23, 24), may explain why some patients with high CXCL8 exhibit poor infection clearance despite robust inflammation. Our data show elevated CXCL8 in recurrence, possibly reflecting such pathogenic hyperinflammation rather than effective recruitment.

According to ROC curve analysis, CXCL8 exhibited excellent evaluation effects on the occurrence and recurrence of BURTIs, which established its potential as an evaluation index of BURTIs in the future. As noted above, sputum culture remains the gold standard for the diagnosis of BURTIs, which is time-sensitive and difficult to achieve large-scale early screening (25). Traditional markers such as C-reactive protein (CRP) (26) and PCT (27) have certain limitations in this population. However, CRP and PCT—though useful for BURTI diagnosis—exhibit reduced sensitivity in immunocompromised elderly patients. In contrast, the association between CXCL8 and neutrophil recruitment provides a more targeted reflection of bacterial load. CXCL8 exhibits favorable evaluation effects on BURTIs, pointing to its possible utility in enhancing the assessment of the occurrence and development of BURTIs. Furthermore, its close relationship with clinical outcomes enables dynamic therapeutic monitoring, which can contribute to earlier diagnosis and support the timely formulation of appropriate interventions, thus ensuring patient prognosis and health. Among them, we chose post-treatment CXCL8 for prognostic assessment owing of its association with BURTI progression. Alterations in CXCL8 levels within the same patient, from pre- to post-treatment, can serve as a dynamic biomarker of therapeutic response. The clinical utility of post-treatment CXCL8 analysis lies in its ability to provide prognostic information and clarify the need for additional therapeutic measures.

Of course, due to the small number of cases in this study, it cannot be excluded that the analysis results may be affected by contingency. Therefore, more cases are needed to validate the results of this study. Similarly, the potential of CXCL8 to serve as a discriminator between bacterial and viral BURTIs warrants further exploration. Besides, the follow-up period needs to be extended to evaluate the relationship between CXCL8 and the long-term prognosis of BURTIs. Finally, we need to carry out *in vitro* trials to confirm the exact mechanism of action of CXCL8 on BURTIs, so as to provide more comprehensive reference and guidance for clinical practice. In the next step, prospective cohort studies will be conducted to simultaneously include patients with viral (such as influenza), bacterial, and mixed infections, and multi-omics analysis (such as transcriptome and metabolome) will be conducted to reveal the dynamic characteristics of CXCL8 in different infection types.

## 5 Conclusion

CXCL8 levels are elevated in elderly patients with BURTIs, showing a correlation with post-treatment recurrence risk. Dynamic monitoring reveals a significant CXCL8 reduction following effective treatment, suggesting its potential to assess therapeutic response. Unlike traditional biomarkers such as CRP/PCT, CXCL8 distinguishes itself through its neutrophil-specific chemotactic activity, enhancing diagnostic specificity in the elderly. These findings position CXCL8 as a promising biomarker for guiding personalized treatment in elderly BURTI patients, though further validation and integration with clinical scores are needed to improve risk stratification.

## Data Availability

The original contributions presented in the study are included in the article/supplementary material, further inquiries can be directed to the corresponding author.
